# Roles of Ion Channels in Oligodendrocyte Precursor Cells: From Physiology to Pathology

**DOI:** 10.3390/ijms26157336

**Published:** 2025-07-29

**Authors:** Jianing Wang, Yu Shen, Ping Liao, Bowen Yang, Ruotian Jiang

**Affiliations:** 1 Department of Anesthesiology, West China Hospital, Sichuan University, Chengdu 610041, China; 2 Laboratory of Anesthesia and Critical Care Medicine, National-Local Joint Engineering Research Center of Translational Medicine of Anesthesiology, West China Hospital, Sichuan University, Chengdu 610041, China

**Keywords:** oligodendrocyte precursor cells, ion channel, central nervous system, neurological diseases

## Abstract

Oligodendrocyte precursor cells (OPCs) are a distinct and dynamic glial population that retain proliferative and migratory capacities throughout life. While traditionally recognized for differentiating into oligodendrocytes (OLs) and generating myelin to support rapid nerve conduction, OPCs are now increasingly appreciated for their diverse and non-canonical roles in the central nervous system (CNS), including direct interactions with neurons. A notable feature of OPCs is their expression of diverse ion channels that orchestrate essential cellular functions, including proliferation, migration, and differentiation. Given their widespread distribution across the CNS, OPCs are increasingly recognized as active contributors to the development and progression of various neurological disorders. This review aims to present a detailed summary of the physiological and pathological functions of ion channels in OPCs, emphasizing their contribution to CNS dysfunction. We further highlight recent advances suggesting that ion channels in OPCs may serve as promising therapeutic targets across a broad range of disorders, including, but not limited to, multiple sclerosis (MS), spinal cord injury, amyotrophic lateral sclerosis (ALS), psychiatric disorders, Alzheimer’s disease (AD), and neuropathic pain (NP). Finally, we discuss emerging therapeutic strategies targeting OPC ion channel function, offering insights into potential future directions in the treatment of CNS diseases.

## 1. Introduction

Oligodendrocyte precursor cells (OPCs) represent a widely distributed population of glial cells within the central nervous system (CNS) [1]. Although they account for only approximately 5–8% of total cells in the CNS, OPCs exhibit remarkable proliferative and migratory capacities and are essential for maintaining CNS homeostasis [2]. OPCs have been traditionally regarded as progenitors that differentiate into mature oligodendrocytes (OLs), the myelin-forming cells of the CNS, responsible for insulating axons and facilitating conduction of rapid and efficient action potentials [3,4]. Recent research continues to highlight the diverse functions of OPCs in various neurological disorders, including multiple sclerosis (MS) [5], Alzheimer’s disease (AD) [6], etc. Beyond their classical role in myelination, OPCs exhibit a range of non-myelinating functions in both health and disease, further underscoring their functional versatility within the CNS.

Accumulating evidence indicates that OPCs can establish functional synaptic contacts with neurons, through which they receive direct synaptic input [7,8]. This form of neuron–glia communication is mediated by a broad array of voltage-gated ion channels and neurotransmitter receptors expressed on the OPC membrane. These ion channels endow them with an intricate electrophysiological characteristic, similar to that of neurons [9]. An increasing body of evidence has demonstrated the functional expression of diverse ion channels throughout the oligodendrocyte lineage [10], highlighting their pivotal roles in key physiological processes, including OPC proliferation [11], migration [12], and differentiation [13].

Despite substantial advances in elucidating the expression profiles and physiological roles of ion channels in OPCs, significant knowledge gaps remain. In particular, the precise expression patterns of specific ion channel subtypes and their involvement in non-canonical OPC functions, such as synaptic integration, neuroimmune modulation, and maintenance of blood–brain barrier (BBB) integrity, are still poorly understood. While much of the current research has focused on demyelinating diseases such as MS, emerging studies suggest that ion channel dysregulation in OPCs may also contribute to a broader range of neurological disorders, including neuropathic pain (NP), amyotrophic lateral sclerosis (ALS), and psychiatric diseases.

This review provides a comprehensive overview of the physiological relevance of ion channels in OPCs. We focus on the roles of voltage-gated sodium (Na^+^), potassium (K^+^), calcium (Ca^2+^), ligand-gated, and other functionally relevant ion channels in regulating the physiological functions of OPCs. These include both classical lineage-related processes, such as proliferation, migration, and differentiation, and non-canonical functions, including synaptic integration, neuroimmune interactions, and modulation of BBB integrity. We also highlight the dynamic changes in ion channel expression that occur during the maturation of OPCs into myelinating OLs. Additionally, we explore the pathological implications of OPC ion channel dysregulation in a range of neurological disorders, including MS, spinal cord injury, ALS, AD, NP, and psychiatric disorders. Finally, we outline emerging therapeutic strategies targeting OPC ion channels for promoting central CNS repair and modulating disease progression.

## 2. Distribution and Functional Role of OPCs in CNS

**Figure 1:**
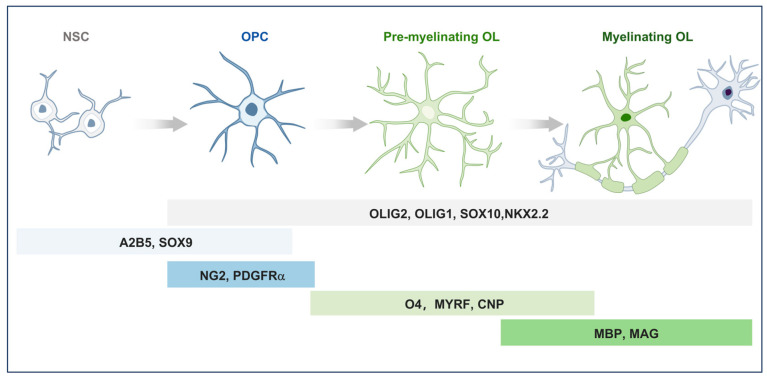
Schematic representation of oligodendrocyte lineage progression and associated molecular markers. Oligodendrocytes arise from neural stem cells (NSCs) through a well-defined lineage progression, with each stage marked by distinct cellular morphology and stage-specific molecular markers. OPCs express cell surface ganglioside epitope (A2B5), SRY-Box Transcription Factor 9 (SOX9), NG2, and PDGFRα. As they differentiate into pre-myelinating OLs, markers such as cell surface markers (O4), Myelin Regulatory Factor (MYRF), and 2′, 3′-cyclic-nucleotide-phosphodiesterase (CNP) are upregulated. Myelinating OLs express myelin-related proteins such as myelin basic protein (MBP) and myelin-associated glycoprotein (MAG).

OPCs are resident stem cells within the CNS that proliferate and migrate to populate it. Despite a notable decrease in oligodendrocyte production concurrent with brain maturation, OPCs remain abundant and retain their capacity for differentiation within the adult brain [14,15]. During CNS myelination, OPCs undergo a tightly regulated sequence of morphological and molecular transitions, ultimately giving rise to myelinating OLs in response to extracellular signals (Figure 1). This differentiation process can be further subdivided into several distinct stages. This multi-step complex process is stringently governed by spatiotemporal-specific transcription factors [16]. During terminal differentiation, OLs extend their processes and progressively ensheath axons with concentric layers of membrane, culminating in the formation of compact myelin [17].

The expression of lineage-specific markers, such as platelet-derived growth factor receptor alpha (PDGFRα), neuron–glial antigen 2 (NG2), and oligodendrocyte transcription factor 2 (Olig2), is widely recognized as characteristic molecular identifiers of OPCs [18]. PDGFRα, the receptor for platelet-derived growth factor (PDGF), serves as a well-established molecular marker for OPC identification, owing to its consistent expression and extensive experimental validation [19]. PDGF, mainly released by neurons and astrocytes, is essential for controlling lineage development of OPCs (Figure 1) [20,21,22,23].

Functionally, OPCs contribute to CNS homeostasis through multiple mechanisms. The primary physiological roles of OPCs encompass differentiation into OLs and subsequent myelination [24]. In addition, OPCs are involved in glial signaling [25], axonal remodeling and plasticity [26], and may also possess the potential to transdifferentiate into other cell types under specific pathological or developmental conditions [27]. The persistence of OPCs within the mature CNS, long after developmental myelination is complete, indicates that these cells fulfill roles beyond conventional myelin production. Although the multifaceted roles of OPCs are increasingly recognized, our understanding of their functions in physiological and pathological conditions remains incomplete.

## 3. Ion Channels in the Physiological Functions of OPCs

**Figure 2:**
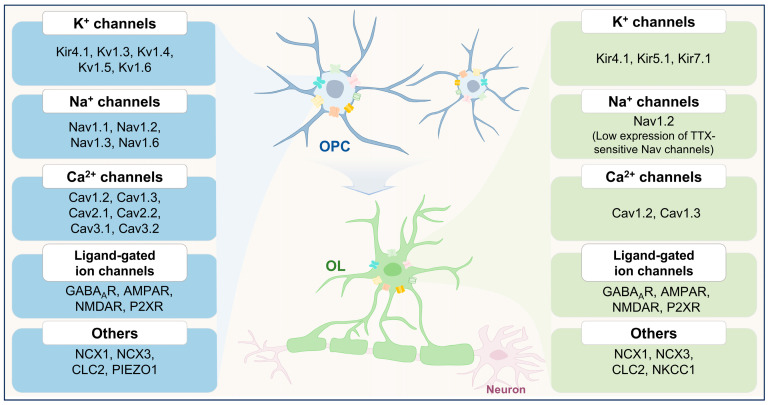
Dynamic changes in ion channel expression during oligodendrocyte lineage progression. Oligodendrocyte precursor cells (OPCs, **top**) and mature oligodendrocytes (OLs, **bottom**) exhibit distinct ion channel expression profiles, reflecting their functional transitions during differentiation. Ion channels are categorized by ion selectivity and are differentially expressed across developmental stages. In OPCs (**left panel**), a broad array of ion channels is present, including potassium channels (Kir4.1, Kv1.3, Kv1.4, Kv1.5, and Kv1.6), sodium channels (Nav1.1, Nav1.2, Nav1.3, and Nav1.6), calcium channels (Cav1.2, Cav1.3, Cav2.1, Cav2.2, Cav3.1, and Cav3.2), ligand-gated channels (GABA_A_R, AMPAR, NMDAR, and P2XR), and other channels such as sodium–calcium exchangers (NCX1 and NCX3) and chloride channels (CLC2). In contrast, mature OLs (**right panel**) display a more restricted ion channel repertoire. TTX-sensitive Nav channels show minimal expression in OLs. These stage-dependent shifts in ion channel expression underscore the functional transformation of OPCs from a proliferative and migratory phenotype to a mature, myelinating state.

OPCs are distinguished from other glial cell types by their capacity to form synapses with neurons and by their expression of diverse voltage-gated ion channels and neurotransmitter receptors, endowing them with unique electrophysiological properties (Figure 2, Table 1) [7,8]. Notably, OPCs express genes encoding ion channels typically considered “neuronal”. This molecular profile confers upon OPCs a complex electrophysiological phenotype that closely resembles that of neurons, in stark contrast to astrocytes and mature OLs [9]. Genes encoding ion channels are essential for neuronal excitability, governing the initiation and propagation of electrical signals [28]. These channels in OPCs allow them to perceive shifts in the membrane potential of surrounding neurons and recognize extracellular neurotransmitters [29], thereby generating and regulating neural electrical activity [30]. The neuron-like nature of OPCs adds complexity and diversity to their role in the CNS.

Importantly, the expression of ion channels in OPCs exhibits dynamic variability and undergoes alterations throughout the process of OPC differentiation into OLs (Figure 2). Like neurons, OPCs also express tetrodotoxin (TTX)-sensitive voltage-gated sodium channels (Nav), α-amino-3-hydroxy-5-methyl-4-isoxazolepropionic acid receptors (AMPARs), and *N*-methyl-_D_-aspartate receptors (NMDARs). The expression of these channels and receptors declines progressively as OPCs differentiate into mature OLs [29]. In addition, voltage-gated potassium channels (Kv), Kv1.3 and Kv1.5, exhibit downregulation during oligodendrocyte lineage progression, suggesting a role in early-stage OPC physiology [31]. Similarly, transcriptomic analyses indicate that the expression of L-type voltage-gated calcium channel (Cav) subunits (e.g., Cav1.2 and Cav1.3) is elevated in OPCs but declines as they mature into myelinating OLs [10]. Notably, members of the selective sodium calcium exchanger (NCX) family also undergo dynamic regulation during OPC differentiation. While NCX1 is downregulated, NCX3 expression is significantly upregulated [32]. These stage-specific alterations in ion channel expression highlight the dynamic electrophysiological remodeling that accompanies OPC-to-OL transition (Figure 2).

**Table 1:** Ion channels involved in the physiological functions of OPCs.

Ion Channel		Functions	Key Findings
**K^+^ Channels**	Kir4.1	Differentiation and myelination	Knockout of *Kcnj10* in OPCs impairs differentiation and myelination [13].
		OPC-neuron interaction	Kir4.1 mediates K^+^ buffering between OPCs and neurons [33].
	Kv1.3, Kv1.4, and Kv1.6	Proliferation	Overexpression of Kv1.3 or Kv1.4 increased OPC proliferation, while Kv1.6 overexpression inhibited cell cycle progression [11].
**Na^+^ Channels**	Nav1.2	Differentiation and myelination	Specific knockout of *SCN2A* disrupts the maturation process of oligodendrocytes [34]. Selective knockout of the *SCN2A* in pre-OLs results in impaired myelin sheath formation [35].
**Ca^2+^ Channels**	Cav1.2	Proliferation, migration, differentiation, and myelination	Cav1.2 deletion in OPCs led to impaired proliferation, migration, differentiation, and myelination [36,37].
	Cav1.2 and Cav1.3	OPC-neuron interaction	Conditional deletion of Cav1.2 and Cav1.3 abolished long-term depression and potentiation [38].
**Ligand-gated ion channels**	GABA_A_Rs	Proliferation, differentiation, and myelination	GABA_A_Rs blockade enhances OPC proliferation, differentiation, and myelination [39].
	AMPARs	Proliferation, migration, differentiation, and myelination	In ex vivo organotypic slice cultures, pharmacological blockade of AMPARs promoted OPC proliferation, differentiation, and myelination [40]. In the developing corpus callosum (P14—21), overexpression of GluA2 suppressed OPC differentiation and enhanced proliferation [41]. GluA2 overexpression in the adult brain (P50—P75), the same manipulation increased OPC proliferation [42]. Activation of AMPARs enhances OPC migration [43,44]. In vivo, in zebrafish, it was demonstrated that mutations in GluA4A reduced migration [45].
	NMDARs	Migration, differentiation, and myelination	NMDAR-mediated calcium influx can direct OPC migration [46]. Activation of NMDARs promotes OPC differentiation [47].
	P2X7Rs	Migration	High concentrations of extracellular ATP can activate P2X7 receptors to promote OPC migration [12].
**Other channels**	CLC-2	Differentiation	CLC-2 inhibition suppresses OPC differentiation [48].
	NKCC1	Myelination	NKCC1 overexpression enhances myelin plasticity [49].
	NCX3	Myelination	Myelination is markedly impaired in NCX3-deficient mice [32].
	Piezo1	Proliferation and differentiation	Piezo1 knockdown in aged OPCs enhances proliferation and differentiation [50].

Summary of key ion channels expressed in OPCs and their roles in proliferation, migration, differentiation, myelination, and neuron–glia interactions.

Elucidating the roles of ion channels in OPCs will deepen our insight into their physiological functions while opening new avenues for investigating their contributions to CNS disorders. Owing to their specialized electrophysiological profiles and dynamic responsiveness to neuronal signals, OPCs are increasingly implicated in the pathophysiology of various CNS disorders, though the precise mechanisms by which they influence disease onset and progression remain to be fully elucidated.

### 3.1. K^+^ Channels

OPCs in brain slices from juvenile animals generally have a resting membrane potential (RMP) of about −80 to −100 mV [9]. This hyperpolarized RMP is closely aligned with the K^+^ equilibrium potential, suggesting that K^+^ channels are the key contributors to the RMP in OPCs. Simultaneously, a wide range of research has detailed the expression of various K^+^ channels in OPCs, including inward rectifying K^+^ channels (Kir), outward rectifying Kv, and two-pore domain K^+^ channels (K2P) [10,51].

Among the ion channels expressed in OPCs, Kir4.1 has emerged as a particularly important regulator of lineage progression. Kir4.1, a major inwardly rectifying K^+^ channel in the CNS, is crucial for establishing the RMP and is broadly expressed across various CNS cell types. RNA-Seq transcriptomic data reveal that Kir4.1 (*Kcnj10*) mRNA is abundantly expressed in OPCs [10]. Specific knockout of Kir 4.1-encoding gene *Kcnj10* in OPCs during CNS development leads to impaired OPC differentiation and defective myelination [13]. Moreover, pronounced vacuolation in white matter tracts has been observed in Kir4.1-deficient models, suggesting that this channel fulfills multiple roles [52]. Mechanistically, Kir4.1 influences intracellular pH and modulates signaling pathways involving GSK3β and SOX10 [53]. Other Kir subtypes, such as Kir5.1 and Kir7.1, may support OL survival and myelin maintenance. [54,55]. Collectively, these findings underscore the multifaceted involvement of Kir4.1 in the developmental trajectory of the oligodendrocyte lineage.

Kv, particularly Kv1.3 and Kv1.4, promote OPC proliferation, while Kv1.6 inhibits mitogen-induced cell cycle progression [11,31]. Interestingly, despite their differential effects on proliferation, the overexpression of Kv1.3, Kv1.4, Kv1.5, or Kv1.6 does not interfere with OPC differentiation [11]. The results indicate that Kv1 channel subtypes specifically regulate OPC cycle dynamics without disrupting their capacity for lineage progression. In addition to their roles in proliferation and differentiation processes, OPCs are highly mobile cells that rely on purinergic G-protein coupled receptor, GPR17, activation for migration function; this activation also enhances outward rectifying sustained K^+^ current [56]. However, the molecular mechanisms underlying these differential effects remain poorly understood. It is plausible that these functional discrepancies are mediated by isoform-specific modulation of signaling pathways and transcription factors involved in OPC lineage progression.

The role of OPCs to differentiate into mature OLs, thereby contributing to CNS myelination, is well-established. Moreover, emerging research supports the notion that these cells possess characteristics beyond being mere progenitors [2]. The potential involvement of ion channels in the myelin-independent roles of OPCs should be simultaneously considered.

The blood vessels of the CNS possess specialized structural and functional properties collectively referred to as the BBB, which precisely regulate the exchange of ions and molecules between the circulation and the CNS. This barrier is primarily maintained by endothelial cells, whose function is tightly regulated through interactions with various vascular, immune, and neural cell types [57]. Emerging evidence suggests that OPCs may also play an active role in maintaining BBB integrity. Within the transient middle cerebral artery occlusion (tMCAO) model, deficiency of the K2P channel, TREK-1, led to a reduction in neuronal death, milder BBB disruption, smaller infarction sizes, diminished immune cell invasion, and enhanced neurological function [58,59]. TREK-1 is expressed predominantly in astrocytes, neurons, and OPCs within the cerebral cortex [60], suggesting that TREK-1 in OPCs is potentially implicated in the maintenance of the BBB. However, the specific contribution of OPC-expressed TREK-1 to BBB function remains unclear. Conditionally, cell-type-specific approaches, such as OPC-targeted deletion or overexpression of TREK-1, are needed to delineate its precise role. These studies could further clarify whether TREK-1 in OPCs contributes to BBB regulation under both physiological and pathological conditions.

The local extracellular concentration of K^+^ ([K^+^]_e_) plays a critical role in maintaining neuronal membrane potential and excitability, which are fundamental for normal neuronal function. In addition to astrocytes, OPCs can also achieve K^+^ buffering through Kir4.1 [33]. A recent study has demonstrated that OLs detect axonal activity by activating the Kir4.1 channel, which in turn regulates metabolite supply to promote axonal health [61]. Conditional deletion of Kir4.1 in oligodendrocyte-lineage cells, including OPCs and OLs, also leads to severe axonal dysfunction and degeneration [62]. In parallel, loss of Kir4.1 in these glial populations also triggers marked behavioral abnormalities, such as neuronal hyperexcitability and seizure [63]. Collectively, these studies underscore the pivotal role of Kir4.1 in oligodendrocyte lineage cells for both maintaining neuronal homeostasis and providing metabolic and functional support to axons.

K^+^ channels significantly influence the proliferation, differentiation, and neural support of OPCs (Table 1). Various types of K^+^ channels show distinct differences in their expression stages and functions, suggesting that the pathways through which ion channels affect OPC physiological functions might vary. It is crucial that we focus more on the downstream pathways of different K^+^ channels that influence the physiological functions of OPCs and OLs. Furthermore, additional comprehensive research is needed to further investigate the involvement of K^+^ ion channels in OPCs’ myelin-independent roles. This will offer a fresh perspective for understanding the physiological functions of cells within the oligodendrocyte lineage and provide research leads for the etiology and treatment of related neurological diseases.

### 3.2. Na^+^ Channels

Accumulating evidence indicates that OPCs express Nav functionally, as evidenced by a TTX-sensitive spike [64,65,66]. During early postnatal brain development, Nav channels contribute substantially to the RMP of OPCs. Importantly, Nav channel expression is largely restricted to immature OPCs and undergoes significant downregulation as these cells differentiate into mature OLs [67,68].

This developmental regulation has been further supported by studies using OL-GFP mice, which express green fluorescent protein (GFP) under the regulatory sequence of the proteolipid protein (PLP) gene. In OL-GFP mice at postnatal days 4–6 (P4–P6), the majority of immature, pre-myelinating oligodendrocytes (pre-OLs) in the corpus callosum lacked detectable inward Na^+^ currents, whereas most OPCs located near the lateral ventricle exhibited robust Nav channel activity [69]. These findings reinforce the concept that the downregulation of Nav channel expression is a key step in the developmental transition from OPCs to pre-OLs.

The previous studies have suggested that the activity of Na^+^ channels during OPCs depolarization may significantly contribute to an increase in proliferation and potentially play a crucial role in CNS development or repair [68]. This hypothesis has received support from more recent studies. Nav1.2, encoded by *SCN2A*, is selectively expressed in immature OLs in the brainstem and cerebellum. Its deletion disrupts oligodendrocyte maturation and myelination [34].

Furthermore, a subset of pre-OLs, which represent an intermediate stage in the OPC differentiation process, exhibit the ability to generate action potentials driven by Nav1.2 channels from postnatal development until early adulthood. These action potentials in pre-OLs are triggered by excitatory signals received from neighboring neurons through glutamate input. Selective knockout of the *SCN2A* in pre-OLs results in altered cellular morphology, diminished axon-oligodendrocyte interactions, and impaired myelin sheath formation [35]. These findings highlight the crucial function of Nav1.2 in regulating oligodendrocyte development and initiating the process of myelination.

Collectively, these findings highlight a critical role for Nav1.2 in coordinating OPC axon-glia communication and the initiation of myelination (Table 1). Given the essential role of *SCN2A* in oligodendrocyte development, its dysregulation may have implications for demyelinating diseases or neurodevelopmental disorders associated with Nav1.2 dysfunction.

### 3.3. Ca^2+^ Channels

Ca^2+^ channels have been shown to regulate many physiological functions of OPCs, including proliferation, migration, differentiation, and myelin formation [69,70,71]. Single-cell RT-PCR and transcriptomic analyses have confirmed the expression of multiple Cav subtypes in OPCs, including L-type channels such as Cav1.2 and Cav1.3 [9].

The results of in vitro experiments demonstrated a positive correlation between the activity of Cav and the migration rate of OPCs. Moreover, it was observed that high K^+^ treatment significantly enhanced the migration rate of OPCs. Conversely, the presence of a Cav inhibitor in the medium exhibited an inverse relationship with OPC migration. Importantly, when both a Cav antagonist and verapamil were introduced to the medium, the K^+^-dependent regulation of OPC speed was abolished [69]. Furthermore, phosphorylation directly modulates the activity of Cav in OPCs. Activation of protein kinase C and tyrosine kinases significantly enhances Cav activity in OPCs, while activation of protein kinase A exerts an opposite effect on its activity. These kinase-mediated effects subsequently impact process dynamics and migration ability in OPCs [72].

Cav1.2 is the most studied subtype. Its knockdown reduces depolarization-induced Ca^2+^ transients, highlighting its predominant role in mediating voltage-dependent Ca^2+^ influx [70]. Cav1.2 targeted deletion in OPCs led to compromised migration and proliferation within the CNS, OL aplasia, significant myelin loss, and reduced axon-glial interaction capability [36]. Simultaneously, conditional knockout of Cav1.2 in OPCs impaired myelin regeneration in the cuprizone (CPZ) model mice, suggesting that Cav1.2 in OPCs is critical for remyelination [37].

While the specific contribution of Cav1.2 to OPC-mediated modulation of neuronal circuits remains to be fully elucidated, ablation of both Cav1.2 and Cav1.3 in OPCs has been shown to disrupt NMDAR-dependent synaptic plasticity, suggesting roles beyond myelination [38].

Collectively, these observations underscore the multifaceted role of Ca^2+^ signaling in OPC biology, not only in lineage progression but also in shaping neuronal network dynamics (Table 1). The involvement of OPC Ca^2+^ channels in activity-dependent processes suggests that OPCs may serve as integrative components of neural circuits, capable of responding to and modulating synaptic activity.

### 3.4. Ligand-Gated Ion Channels

In contrast to other glial cells, OPCs are capable of receiving direct synaptic input from neurons, emphasizing their dynamic interaction with the neuronal network [1,73]. The expression of various ligand-gated ion channels (LGICs) in both OPCs and OLs provides the structural and molecular foundation for this bidirectional communication. Among these, AMPAR and NMDARs mediate excitatory glutamatergic inputs, while GABA_A_ receptors (GABA_A_Rs) and P2X receptors (P2XRs) contribute to inhibitory and purinergic signaling, respectively.

#### 3.4.1. GABA_A_Rs

In ex vivo cerebral cortical slice cultures, endogenous γ-aminobutyric acid (GABA) release has been shown to suppress OPC proliferation, increase cell death, and impair myelination via activation of GABA_A_Rs [39]. These findings highlight a broader role for GABAergic interneurons in regulating myelin dynamics. By altering OPC development, GABAergic activity can ultimately influence the speed of action potential conduction.

OLs are known to express ionotropic receptors for GABA, glutamate, and adenosine triphosphate (ATP). Nevertheless, when cultured in isolation, OLs exhibit drastically reduced receptor expression and diminished GABA_A_Rs. In contrast, the amplitude of the GABA response in OLs cocultured with dorsal root ganglion neurons is maintained and remains stable [74]. These results emphasize the essential role of neuron-derived signals, particularly from inhibitory interneurons, in maintaining GABA_A_R-mediated signaling and supporting oligodendrocyte maturation and myelination [75]. The functional importance of GABA_A_R signaling in vivo is further supported by studies in a mouse model of chronic neonatal hypoxia. In this model, the loss of GABA_A_R-mediated synaptic input to OPCs disrupted differentiation and myelination, whereas enhancing GABA availability promoted oligodendrocyte differentiation and myelin production [76]. Mechanistically, GABA_A_R activation in OPCs not only induces chloride flux but also interacts with other ionotropic signaling pathways. In the hippocampus, functional synapses are formed between OPCs and GABAergic interneurons through direct synaptic inputs. The GABA-induced Cl^-^ flux may impact oligodendrocyte development by modulating glutamatergic signaling in OPCs [8].

#### 3.4.2. AMPARs

Glutamate exerts its biological effects through ionotropic glutamate receptors and metabotropic glutamate receptors. Ionotropic glutamate receptors are categorized into three main subtypes: AMPARs, NMDARs, and kainate receptors (KARs), based on their pharmacological characteristics and selective agonists [77].

The central structure of the AMPAR complex is a tetramer composed of GluA1, GluA2, GluA3, and GluA4 subunits [75]. Despite widespread expression of AMPARs in OPCs, their precise functional role remains controversial, as findings from various experimental models have been inconsistent.

Activation of AMPARs has been reported to interfere with OPC proliferation and lineage progression in early in vitro models [78], suggesting a suppressive role of glutamatergic signaling in oligodendroglia development. In cerebellar organotypic slice cultures, pharmacological blockade of AMPARs promoted OPC proliferation and differentiation but impaired morphological maturation and myelination [40]. In vivo genetic studies further complicate the picture: triple knockout of GluA2, GluA3, and GluA4 does not affect OPC proliferation, but compromises the survival of pre-OLs, resulting in a reduced mature OL population without significantly impairing myelin synthesis per se [79]. In the developing corpus callosum (P14—21), retroviral overexpression of GluA2, thereby altering AMPAR Ca^2+^ permeability, suppressed OPC differentiation and enhanced proliferation [41]. However, when the same manipulation was applied during earlier postnatal stages (P3—P15), no measurable changes were observed in OPC dynamics or myelination across multiple brain regions, including the cerebral cortex, corpus callosum, and external capsule. Interestingly, in the adult brain (P50—P75), GluA2 overexpression led to a renewed increase in OPC proliferation, suggesting a developmental stage-dependent effect of AMPAR signaling on OPCs [42]. These inconsistencies may arise from variations in experimental approaches (e.g., in vitro vs. in vivo), the specific brain regions studied (such as the cerebellum, cerebral cortex, corpus callosum, and external capsule), and the developmental stage of the animals (developing vs. adult brain). Collectively, these findings underscore the region- and age-specific heterogeneity in AMPA receptor-mediated modulation of OPC physiology.

These seemingly contradictory findings likely reflect the intrinsic heterogeneity of OPCs and their context-dependent responses to neuronal activity. The regional and temporal variability in AMPAR function may represent a finely tuned mechanism by which OPCs integrate excitatory synaptic input to regulate their behavior in accordance with CNS developmental or pathological demands. A more detailed understanding of the subunit-specific and region-specific roles of AMPARs in OPCs is therefore essential. Such insights will not only help clarify the physiological basis of OPC heterogeneity but also provide a framework for interpreting region-specific vulnerabilities or adaptations of OPCs in demyelinating and other CNS diseases.

Compared to the debated roles of AMPARs in OPC proliferation and differentiation, their involvement in OPC migration appears to be more consistently supported across various experimental systems. Glutamate has been identified as a critical long-range guidance cue for OPC migration. In both organotypic cerebellar slice cultures and cell models, AMPARs activation leads to increased intracellular Ca^2+^ signaling, which enhances OPC migration [43,44]. This mechanism has also been confirmed in vivo. This mechanism has been validated in vivo using time-lapse imaging in zebrafish, where GluA4A (*gria4α*) mutations impair glutamate sensing in OPCs and reduce their migratory capacity. Interestingly, this migratory defect can be rescued by treatment with (±)-Bay K 8644, a Cav agonist, suggesting that Cav function downstream of AMPAR-mediated Ca^2+^ influx to regulate OPC migration [45].

Alterations in AMPAR subunits can significantly affect key cellular processes such as migration, proliferation, differentiation, and myelination. Given the differential contributions of individual subunits, targeting specific AMPAR subunits represents a promising direction for modulating OPC function under both physiological and pathological conditions.

Despite growing interest, the exact contributions of AMPARs to OPC biology remain only partially elucidated. The functional impact of AMPAR signaling may vary depending on brain region, developmental stage, and experimental model. To refine our understanding, future investigations should dissect the subunit-specific roles of AMPARs within distinct CNS compartments and temporal windows. To refine our understanding, future investigations should dissect the subunit-specific roles of AMPARs within distinct CNS compartments and temporal windows.

#### 3.4.3. NMDARs

NMDARs, a key class of ionotropic glutamate receptors, play indispensable roles in both the maturation and functional regulation of the CNS. In neurons, NMDARs are key regulators of neural progenitor cell migration, differentiation, and dendritic morphogenesis [77]. Increasing evidence indicates that NMDARs are also functionally expressed in OPCs, where they contribute to multiple aspects of OPC development. In vitro studies using purified rat OPCs revealed that activation of NMDARs promotes oligodendrocyte lineage progression by upregulating the expression of myelin-associated proteins and increasing arborization during the transition to mature OLs [47]. Pharmacological inhibition of NMDARs using the specific antagonist MK801 significantly delays OPC differentiation and remyelination in the CPZ demyelination model, further supporting their functional role in myelin repair [47]. In addition, NMDAR-mediated Ca^2+^ influx can activate the Tiam1/Rac1 signaling pathway, thereby directing OPC migration in a Ca^2+^-dependent manner [46].

#### 3.4.4. P2X7Rs

Similarly to glutamate, extracellular ATP functions as an excitatory signaling molecule within the CNS by activating ionotropic purinergic receptors, particularly those of the P2X subfamily. P2X receptors are ATP-gated ion channels that mediate the efflux of intracellular K^+^ and the influx of extracellular Na^+^ and Ca^2+^, ultimately leading to membrane depolarization [80]. OPCs express multiple P2XRs, including P2X1R, P2X2R, P2X3R, P2X4R, and P2X7R, which can modulate OPC function via intracellular Ca^2+^ signaling [81]. Nevertheless, the specific role of P2X receptors in the biology of OPCs is still not well comprehended. Among them, P2X7Rs have received limited but growing attention. Existing studies suggest that P2X7Rs may participate in the cellular response to OPC injury. For instance, elevated extracellular ATP levels have been shown to activate P2X7Rs, triggering downstream Fyn kinase signaling that facilitates migration of OPCs [12]. In a hypoxia-ischemia model, the expression of P2X7Rs was observed to decrease in OPCs, indicating a possible involvement in injury adaptation or repair processes [82].

### 3.5. Other Channels

The functional expression of voltage-gated chloride channel-2 (CLC-2) was observed in both OPCs and OLs, while the inhibition of CLC-2 resulted in the suppression of OPC differentiation into OLs [48]. The aforementioned study provides additional evidence that the investigation of chloride channels’ involvement in the normal physiological function of OPCs necessitates further research. The functional expression of the Na^+^-K^+^-Cl^−^-co-transporter 1 (NKCC1) was observed in OLs. Overexpression of NKCC1 in these cells has the potential to regulate plasticity of myelinated fibers, thereby facilitating long-term potentiation and enhancing learning [49]. At the same time, NCX3 plays distinct roles in oligodendrocyte maturation. NCX3-knockout mice show a reduction in myelination [32]. Supporting this, recent evidence has shown that NCX promotes calcium influx in developing OPCs and facilitates the synthesis of MBP [83]. Notably, neuronal activity-induced elevation of [K^+^]ₑ may modulate NCX activity, thereby converting extracellular signals into intracellular ionic responses and ultimately regulating OPC function. The mechanosensitive ion channel PIEZO plays a critical role in regulating cell density and stem cell activation by mediating the influx of secondary messengers, particularly Ca^2+^. Notably, PIEZO1 exhibits enriched expression in OPCs. In aged rat OPCs, knockdown of Piezo1 was shown to affect intracellular Ca^2+^ dynamics, thereby promoting both proliferation and differentiation of OPCs [50]. This finding highlights the importance of mechanosensitive ion channels in enabling OPCs to sense and respond to changes in the CNS microenvironment. Through the modulation of intracellular signaling cascades, channels such as PIEZO1 may serve as key molecular transducers that convert mechanical cues into adaptive cellular responses, ultimately influencing OPC behavior and myelin repair capacity.

OPCs interact with microglia, the resident immune cells of the CNS, through a dynamic bidirectional regulatory crosstalk. OPCs are necessary for maintaining immune balance in the CNS through transforming growth factor-β2 (TGF-β2)-TGF-β type II receptor (TGFBR2)-CX3C chemokine receptor 1 (CX3CR1) signaling, which inhibits microglia activation. Mice lacking OPCs exhibit downregulation of microglia-specific gene expression and an exaggerated neuroinflammatory response following exposure to the endotoxin lipopolysaccharide (LPS) [84]. Experimental co-culture models further support this interaction; when OPCs are cultured with wild-type microglia following oxygen-glucose deprivation (OGD), they exhibit increased apoptotic activity and impaired proliferation and maturation. In contrast, when OPCs are co-cultured with microglia that lack Hv1, they experience reduced apoptosis, enhanced proliferation, and improved differentiation [85]. Hv1 is essential for modulating microglial activation and reactive oxygen species (ROS) production [86]. Although direct evidence regarding the involvement of ion channels expressed in OPCs in immune regulation remains limited, it is highly plausible that these channels contribute to modulating microglial activity under both physiological and pathological conditions. Further investigation is needed to clarify how ion channels expressed by OPCs modulate microglial activity and contribute to neuroinflammatory processes.

**Figure 3:**
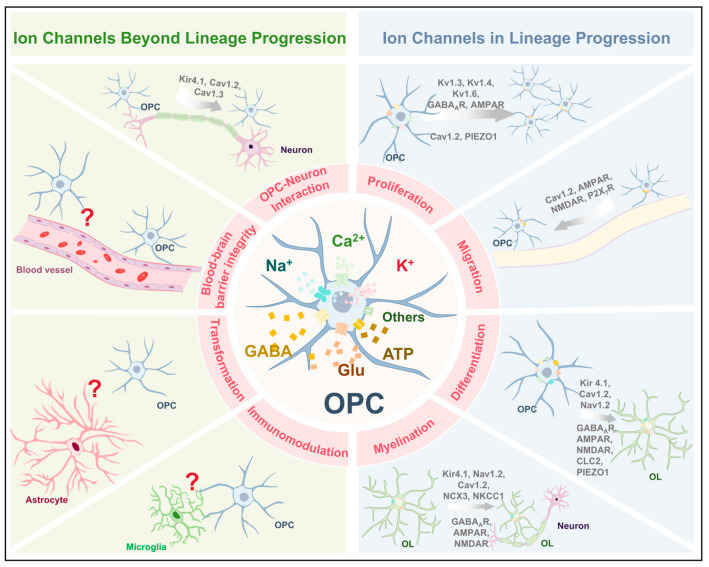
Ion channels involved in the physiological functions of OPCs. Ion channels mediate a broad range of physiological functions in OPCs, which can be broadly categorized into two major domains: those involved in oligodendrocyte lineage progression (**right**, **blue panels**) and those extending beyond classical lineage functions (**left**, **green panels**). On the right, ion channel activity orchestrates key developmental processes, including OPC proliferation, migration, differentiation, and myelination. Representative ion channels implicated in these processes include voltage-gated calcium channels (Cav1.2 and Cav1.3), voltage-gated potassium channels (Kv1.3, Kv1.4, and Kv1.6), inwardly rectifying potassium channels (Kir4.1), chloride channels (CLC-2), sodium channels (Nav1.2), and ligand-gated receptors (GABA_A_R, AMPAR, NMDAR, and P2X7R). On the left, emerging evidence suggests that OPCs also engage in non-canonical roles via ion channel-dependent mechanisms, including interactions with neurons, astrocytes, microglia, and the vasculature. These interactions may modulate synaptic activity, neuroimmune signaling, and blood–brain barrier (BBB) integrity. Ion channels such as Kir4.1, Cav1.2, and Cav1.3 have been implicated in these processes, whereas others remain unidentified or poorly characterized (indicated by question marks). OPCs are not merely progenitors of myelinating oligodendrocytes, but also dynamic regulators of CNS homeostasis and pathology through ion channel-mediated mechanisms.

A large number of studies have characterized the expression patterns of various ion channels on the surface of OPCs, highlighting their pivotal roles in both canonical and non-canonical cellular functions (Figure 3). Ion channels are critically involved in regulating key stages of oligodendrocyte lineage progression, including proliferation, migration, differentiation, and myelination. In addition, they contribute to non-canonical functions such as neuron–glia communication. However, the potential involvement of ion channels in emerging roles of OPCs, such as immunomodulation and maintenance of BBB integrity, remains poorly understood and warrants further investigation. Notably, the expression profiles of multiple ion channel types undergo dynamic changes as OPCs differentiate into mature OLs, underscoring their importance as regulatory elements in OPC physiology. Modulating the activity of these channels could be a promising therapeutic approach to improve remyelination and functional recovery in cases of neural injury and neurodegenerative diseases.

## 4. Pathological Roles of Ion Channels in OPCs

Glial cells are now widely acknowledged as pivotal regulators in the development and progression of nearly all neurological conditions. Within this diverse group, oligodendrocyte lineage cells, particularly OPCs, have emerged as key contributors to a range of CNS diseases. Ion channels, including both ligand-gated and voltage-gated types, are well-established therapeutic targets and are among the most frequently approved drug targets by the U.S. Food and Drug Administration (FDA) [87]. As discussed previously, OPCs express a broad spectrum of ion channels that are not only electrophysiologically active but also integral to the regulation of essential cellular behaviors. Due to their essential role in preserving CNS homeostasis, growing evidence implicates OPC-expressed ion channels in the progression of a variety of neurological disorders (Table 2). These channels function not only as molecular indicators of pathological conditions but also as promising targets for therapeutic intervention.

### 4.1. Multiple Sclerosis

MS is a chronic autoimmune disorder marked by CNS inflammation and progressive demyelination. In individuals with MS, remyelination is primarily driven by OLs [96]. The recruitment and differentiation of OPCs within MS lesions are also critical steps in the remyelination process [97]. Supporting this, a recent study has demonstrated a reduction in OPCs and an upregulation of myelination-related genes in mature OLs within the MS [5]. Consequently, OPCs have also emerged as a significant focus in the research on the pathogenesis and treatment of MS diseases.

Autoantibodies targeting glial ion channels have attracted increasing interest as potential biomarkers in demyelinating diseases. Among these, Kir4.1 has emerged as a candidate antigen. Initial serological studies identified Kir4.1-specific IgG in approximately 47% of MS patients, while it was virtually absent in individuals with other neurological conditions and healthy controls (HCs) [98]. In a follow-up investigation, the same group reported similar antibodies in over half of pediatric patients diagnosed with acquired demyelinating disease (ADD), while such antibodies were absent in individuals with non-demyelinating neurological disorders and HCs [99]. Although these early observations suggested a possible pathogenic role for Kir4.1 autoimmunity in both adult MS and pediatric ADD, later investigations failed to consistently replicate these findings, raising concerns about the specificity, reproducibility, and diagnostic value of anti-Kir4.1 antibodies [100,101,102].

Recent studies have also revealed that Kir4.1 expression in oligodendrocytes undergoes dynamic modulation during both demyelination and subsequent remyelination. Notably, in acute and chronically active demyelinated lesions of MS, Kir4.1 immunoreactivity is selectively diminished in OLs, while its reappearance is observed in remyelinating regions [88]. To elucidate the functional significance of Kir4.1 in OLs, a conditional knockout mouse model targeting *Kcnj10* specifically in OLs was employed. The results demonstrated that OL-expressed Kir4.1 is essential for maintaining white matter integrity during chronic demyelinating injury [62].

Additionally, in the animal model, Nav1.6 exhibited aggregation at the node of myelin regeneration in the focal demyelinating lesion model [103]. Furthermore, both Nav1.2 and Nav1.6 exhibit elevated expression levels at sites of axonal demyelination in the experimental autoimmune encephalomyelitis (EAE) model, a murine model of MS [90]. Notably, treatment with nimodipine, a Cav antagonist, was reported to mitigate demyelination and markedly improve neurological outcomes in EAE. These effects may be mediated, at least in part, by the modulation of intracellular Ca^2+^ dynamics in OPCs, as well as by influencing the expression of specific microRNAs implicated in OPC differentiation and myelin formation [104]. These results underscore the potential of Ca^2+^ channels as promising therapeutic targets in demyelinating diseases such as MS [105]. Piezo1 levels are significantly downregulated in the white matter regions of brains with MS pathology, and in vitro evidence suggests that this mechanosensitive ion channel is critically involved in regulating oligodendrocyte proliferation and migration [89]. Given its critical role in oligodendrocyte development and its downregulation in MS, Piezo1 represents a promising therapeutic target for promoting remyelination and restoring white matter integrity in demyelinating diseases. The NMDAR antagonist, memantine, exhibits a reduction in neurological disability in an EAE rat model by antagonizing the effects of glutamate released in the inflammatory lesion. This drug has the potential to mitigate myelin damage [106,107]. In the EAE mouse model, NCX3 expression was markedly increased during the chronic phase of the disease and was found to be co-localized with OPCs. Genetic ablation of NCX3 led to defective myelination by oligodendrocytes and aggravated neurological deficits in EAE [91].

Although the specificity of Kir4.1 autoantibodies in patients with MS remains a subject of debate, accumulating evidence from animal models points to a potential pathogenic role of Kir4.1 dysfunction. Evidence from experimental models indicates that Kir4.1 expression in oligodendrocyte lineage cells plays a pivotal role in the pathogenesis of EAE models, implicating this channel as a key modulator of demyelination and remyelination. In addition, other ion channels expressed in OPCs, such as Piezo1 and NCX3, have been identified as contributors to disease dynamics in various MS models, further supporting the therapeutic relevance of OPC ion channels in demyelinating conditions.

Beyond their canonical roles in myelination, cells of the oligodendrocyte lineage are increasingly recognized as active participants in CNS inflammation [108] and neuroprotection [109] during MS. Notably, ion channels such as Kir4.1 have been shown to modulate these processes, suggesting that their functions in OPCs extend beyond myelin regulation. These non-canonical roles imply that OPC ion channels may influence MS disease progression not only through intrinsic lineage-specific mechanisms but also via intercellular interactions with immune cells, neurons, and vascular components. Elucidating how ion channels mediate such cross-cellular communication represents a promising avenue for uncovering novel pathophysiological mechanisms and developing targeted therapeutic strategies.

### 4.2. Traumatic Injury of Central Nervous System

The occurrence of traumatic central nervous system injuries (TCNSIs), including spinal cord injury (SCI) and traumatic brain injury (TBI), can lead to profound and catastrophic consequences. TBI impacts people of all age groups and is a main contributor to both mortality and disability worldwide. It is estimated that roughly 10 million individuals are affected globally each year [110]. The mortality rate can reach as high as 13 cases per 100,000 individuals [111].

Traumatic injuries to the CNS initiate persistent apoptosis of mature oligodendrocytes and elicit a rapid response from OPCs, leading to morphological alterations, enhanced proliferation, and directed migration toward the injury site [112]. This reactive response is accompanied by increased expression of NG2 and other glial markers at and around the lesion site, indicating the active involvement of OPCs in injury-induced tissue remodeling [113,114]. In response to CNS injury, OPCs demonstrate rapid activation, surpassed only by microglia [115,116]. Their reactivity contributes to the creation of glial scars and the development of a fibrotic barrier.

A short-lived increase in the proliferative activity of resident OPCs has been observed in the central fluid percussion injury (cFPI) model, a widely used experimental paradigm of TBI that induces diffuse axonal damage [117]. Aging has been identified as a detrimental prognostic factor of functional recovery and prognosis following TBI. In aged mice, the proliferative and differentiative capacity of OPCs is significantly diminished, resulting in reduced generation of new oligodendrocytes post-injury. These results highlight the crucial role of OPCs in determining the prognosis and regenerative capacity following TBI [118].

Recent studies have identified several pharmacological agents with the capacity to promote OPC maturation and facilitate neurological recovery following TCSNIs. For instance, a composite formulation of co-ultramicronized N-palmitoylethanolamine (PEA) and luteolin has been shown to promote OPC maturation and significantly improve motor performance in animal models of SCI or TBI [119,120]. Similarly, recombinant human erythropoietin (rhEPO), a multifunctional neuroprotective cytokine whose receptors are expressed throughout all stages of oligodendrocyte lineage cell differentiation, has been shown to enhance OPC proliferation and myelin formation in mice subjected to controlled cortical impact (CCI) injury [121].

The administration of Amantadine, an NMDAR antagonist and indirect dopamine agonist, has been associated with accelerated functional improvements during the treatment phase in individuals suffering from post-traumatic disorders of consciousness. Although the precise mechanisms driving this therapeutic effect remain to be fully elucidated [122,123], accumulating evidence from both ex vivo and in vitro models indicates that pharmacological blockade of NMDARs negatively affects OPC motility, lineage progression, and remyelination potential [47]. Remarkably, OPCs seem capable of maintaining their structural and functional characteristics even in the absence of NMDAR-mediated signaling, likely via compensatory enhancement of Ca^2+^-permeable AMPA receptor activity [124]. Collectively, these observations underscore the intricate regulatory relationship between glutamatergic neurotransmission and OPC-mediated repair mechanisms within the injured CNS. Understanding this dynamic may provide therapeutic insight into how excitatory neurotransmission modulates OPC responses in neurotrauma contexts.

The specific alterations to NMDARs on the surface of OPCs in nerve injury and potential therapeutic targets still require verification. In addition to glutamatergic receptors, Cav has also been implicated in white matter pathology following CNS trauma. In an in vitro model of isolated segmental spinal cord compression, Cav-mediated Ca^2+^ influx in astrocytes and oligodendrocytes was associated with impaired axonal conduction and secondary degeneration [92]. These findings underscore the potential of targeting Cav to preserve white matter integrity and promote remyelination via OPC modulation.

Currently, there is limited direct evidence supporting the efficacy of targeting ion channels specifically on OPCs for the treatment of TCNSIs. Nevertheless, modulating OPC proliferation and differentiation through ion channel regulation holds significant therapeutic potential. Encouragingly, some pharmacological agents, such as amantadine, have demonstrated beneficial effects in TCSNI models, potentially exerting their actions through ion channels expressed in OPCs. However, the clinical utility of such agents is limited by reports of serious adverse effects, including ophthalmic vein thrombosis, pulmonary embolism, and myocarditis [125,126]. These concerns underscore the need to identify and validate ion channels in OPCs as bona fide therapeutic targets, thereby enabling the development of more selective and safer compounds. Therefore, future studies should prioritize the systematic investigation of the spatiotemporal expression and functional roles of ion channels in OPCs within the context of TCSNIs.

### 4.3. Neurodegenerative Disease

#### 4.3.1. Amyotrophic Lateral Sclerosis

ALS is a fatal neurodegenerative disease of the CNS, characterized by the progressive degeneration of upper and lower motor neurons. This leads to muscle weakness and atrophy affecting voluntary functions such as movement, speech, swallowing, and breathing, and results in a broad range of clinical presentations [127]. Although several pathogenic mechanisms have been proposed to elucidate the selective loss of motor neurons in ALS, accumulating evidence suggests that neurodegeneration is not the sole contributor. Dysfunction of non-neuronal cells, particularly oligodendrocytes in the CNS, has been increasingly implicated in driving disease progression [128,129].

OPCs play an essential role in maintaining axonal integrity and function. Under physiological conditions, the Kir4.1 channel expressed by oligodendrocytes mediates the clearance of excess extracellular K^+^, thereby maintaining K^+^ homeostasis and regulating neuronal excitability [33]. Under physiological conditions. ALS is not a typical demyelinating disease. However, recent observations in the *SOD1^G93A^* rat model have revealed a downregulation and impaired functionality of Kir4.1 in oligodendrocytes, suggesting that previously overlooked ion channels within this glial population may contribute to ALS development [93].

Furthermore, in the early symptomatic stage, MBP: mtSOD1 zebrafish exhibited behavioral, cognitive, and motor impairments, resembling the pathological features observed in the *SOD1^G93A^* mouse model of ALS. These deficits were associated with reduced axonal conduction capacity. Notably, administration of the pan Kv inhibitor 4-aminopyridine effectively mitigated this phenotype [130]. In patients with MS, 4-aminopyridine improves motor function by restoring conduction in demyelinated axons through blockade of Kv [131]. In the context of ALS, 4-aminopyridine may exert therapeutic benefits by enhancing axonal conduction; however, whether it directly influences OPCs remains unclear. In parallel, in vitro studies using human induced pluripotent stem cell (hiPSC)-derived OPCs carrying *fused in sarcoma* (FUS) gene mutations demonstrate aberrant Ca^2+^ signaling, further implicating OPC dysfunction in the complex molecular mechanisms underlying ALS [132]. This finding further implicates the potential involvement of OPCs in the pathogenesis of ALS.

In conclusion, accumulating evidence underscores the critical involvement of oligodendrocyte lineage dysfunction in the pathogenesis of ALS. However, direct evidence linking ion channel dysregulation in OPCs to ALS progression remains limited. Given that impaired axonal conduction is a hallmark of ALS pathology, future studies should aim to characterize, in situ, the roles and alterations of ion channels, beyond Kir4.1, in both OPCs and OLs, particularly in the context of maintaining axonal function. Such investigations may help identify novel therapeutic targets and provide deeper mechanistic insights into glial contributions to ALS pathophysiology.

#### 4.3.2. Alzheimer’s Disease

AD is a progressive neurodegenerative condition marked by cognitive decline and dementia, and imposing a substantial burden on patients, families, and healthcare systems. In the United States alone, approximately 6.7 million individuals aged 65 and older are affected by AD [133]. Despite its high prevalence and significant societal burden, no current therapeutic interventions have been shown to halt or reverse disease progression [134].

At the pathological level, AD is distinguished by the accumulation of amyloid beta (Aβ) plaques and tau neurofibrillary tangles in the brain, which ultimately lead to widespread neuronal damage and degeneration [135]. However, the available evidence suggests that these pathological changes may also affect the oligodendrocyte lineage, with a potential predominance of OLs and their progenitors [136,137]. Given the pivotal role of OPCs in both myelin formation and regeneration, any alterations in their quantity or functionality may compromise the integrity of myelin and thereby contribute to the pathogenesis of AD.

In the initial stages of AD, damage to white matter is apparent [138], accompanied by a significant reduction in Olig2-expressing cells within the human postmortem AD tissue [139]. While differentiating into OLs, OPCs may also contribute to modifications in the oligodendrocyte lineage and myelin damage, as evidenced by observed alterations in NG2 immunoreactivity within the brains of individuals with AD [140]. Moreover, animal experiments conducted on 3× Tg-AD mice suggest that the destruction of OPCs serves as an early pathological indicator for AD and potentially accelerates both cognitive decline and loss of myelin [6]. Similarly, in the APP/PS1 mouse model, characterized by chronic Aβ deposition, restoration of myelin integrity was associated with increased OPC proliferation and differentiation [139,141]. Therefore, in addition to the loss of oligodendrocytes and demyelination, the pathological changes observed in AD may also be associated with impaired mechanisms mediated by OPCs for myelin repair. The findings suggest that alterations in OPCs could potentially contribute to the progression of AD pathology.

The loss of myelin and alterations in the quantity and functionality of OLs are notable features observed in patients with AD as well as AD mouse models. OPCs play a role in this process, and their early destruction serves as a pathological indicator for AD. Recent research has emphasized the possible role of ion channels in modulating OPC function in the context of AD. Piezo1 is selectively upregulated in microglia associated with Aβ plaques. Pharmacological activation of microglial Piezo1 effectively reduces brain Aβ burden and improves cognitive impairment in 5× FAD mice [142]. The expression of Piezo1 has also been noted in both human OLs and OPCs. Inhibition of Piezo1 enhances the proliferation and migration of OPCs, contrasting with microglia [89]. Targeted inhibition of Piezo1 in aging animals greatly enhances the proliferation and differentiation of OPCs [50]. It is further speculated that regulation of Piezo1 activity in OPCs may promote their proliferation and differentiation, enhance myelin regeneration, and thereby potentially alleviate symptoms associated with AD.

Pharmacological agents traditionally used in AD treatment may also exert beneficial effects on oligodendrocyte lineage cells. The acetylcholinesterase inhibitor (AChEI) donepezil is widely employed as a safe and efficacious long-term therapeutic intervention for AD. It exerts its therapeutic effects by reversibly inhibiting AChE, thereby resulting in increased levels of acetylcholine (Ach) within the brain. Furthermore, it has been observed that donepezil significantly promotes the differentiation of OPCs into mature OLs, consequently facilitating myelin formation. Importantly, this effect is independent of its AChE inhibition [143]. Furthermore, administration of mecamylamine, a nicotinic acetylcholine receptor (nAChR) antagonist, partially attenuates the increase in OL numbers observed following donepezil treatment [144], suggesting that nAChR signaling may contribute to donepezil-induced OPC differentiation. A significant aspect of oligodendrocyte pathology in AD involves the disruption of glutamate signaling and Ca^2+^ homeostasis [145]. The prolonged activation of NMDAR results in the demise of oligodendrocytes and impairment of myelin integrity [146]. One of the FDA-approved drugs, memantine, protects the brain from excessive levels of a neurotransmitter called glutamate [133]. The dysregulation of oligodendrocyte glutamate signaling can be mitigated by memantine, which exerts partial blockade on NMDARs [147]. The results of these studies indicate that using NMDAR blockers to target oligodendrocytes could potentially be a crucial component in the treatment strategy for AD patients [148].

Collectively, these findings underscore the critical involvement of OPCs and their associated ion channels in the pathophysiology of AD. A growing body of research has demonstrated that ion channels expressed by OPCs are essential for maintaining their homeostatic and functional integrity. However, their precise pathological roles during the progression of AD remain largely undefined. Although emerging evidence suggests that specific ion channels, such as Piezo1, may contribute to AD pathogenesis via OPC-mediated mechanisms, there is still a lack of reliable preclinical or clinical data evaluating the therapeutic efficacy of targeting these channels.

Furthermore, considering the widespread neuronal degeneration observed in AD, how alterations in OPC-expressed ion channels affect their neuro-supportive functions throughout disease progression remains poorly understood. Thus, the contribution of OPCs and the ion channels they express to AD pathophysiology has yet to be fully elucidated and warrants further investigation. Future studies focusing on the spatiotemporal regulation of OPC ion channels and their interactions with neuronal and glial networks may offer novel insights into AD pathogenesis and identify new therapeutic targets.

### 4.4. Psychiatric Disorders

Psychiatric disorders significantly impact medical, societal, and economic aspects, becoming the leading cause of disability worldwide [149]. Increasing evidence suggests that abnormalities in white matter integrity and myelination are common features underlying the pathophysiology of major psychiatric disorders, including major depressive disorder (MDD), schizophrenia, and bipolar disorder. However, the role of OPCs in these disorders has remained relatively underexplored [116].

Postmortem studies of individuals with schizophrenia, bipolar disorder, and MDD have observed structural alterations in oligodendrocytes and myelinated nerve fibers within the brain [150,151,152,153]. Ketamine has been shown to exert rapid and sustained antidepressant effects in rodent models. Ketamine promotes OPC differentiation and myelination in mice exposed to chronic social defeat stress (CSDS) by targeting AMPARs in OPCs, which helps counteract the myelin loss and depression-like behaviors induced by CSDS [95]. This finding suggests that AMPAR-mediated modulation of OPCs may contribute to the therapeutic effects of ketamine in stress-related disorders. In a single-nucleus transcriptomics analysis of postmortem brain tissue from patients with MDD, deep-layer excitatory neurons and OPCs collectively account for nearly half (47%) of all observed changes in gene expression [154]. In a recent study, a mutation in the TMEM63A gene, which encodes a mechanosensitive cation channel, was identified in a patient with autism spectrum disorder and attention-deficit/hyperactivity disorder, who showed significant hypomyelination on brain MR. In *Tmem63a^−^/^−^* mice, the absence of TMEM63A was shown to affect OPC differentiation, pointing to its essential function in developmental myelination [94]. Together, these findings implicate OPC dysfunction as a potential contributor to the neurobiological mechanisms underlying diverse psychiatric disorders and highlight its therapeutic relevance.

Although there are presently no efficacious antipsychotic drugs designed for ion channel targeting of OPCs, it is noteworthy that current antipsychotic medications have the potential to improve dysfunction in OPCs, thus enhancing therapeutic effectiveness for individuals with schizophrenia. The negative effect of Cuprizone on the differentiation of OPCs can be greatly mitigated by Clozapine and Quetiapine [155]. Concurrently, clozapine enhanced the energy provision and myelin synthesis of cultured oligodendrocytes [156]. The administration of haloperidol induces activation of quiescent OPCs in the adult mouse brain [157]. In a posttraumatic stress disorder (PTSD) mouse model, treatment with fluoxetine was shown to activate the Wnt/β-catenin signaling pathway in OPCs, leading to the restoration of normal differentiation and process outgrowth [158]. These findings further support the hypothesis that OPCs may be responsive to pharmacological modulation and could serve as a cellular target for psychiatric drug development.

Despite these promising observations, direct evidence linking specific ion channels in OPCs to the pathogenesis of psychiatric disorders remains limited. Nevertheless, it is well established that OPC dysfunction and myelin abnormalities are key pathological features in various psychiatric conditions, including MDD. Furthermore, several psychotropic medications, such as clozapine and quetiapine, have been shown to promote OPC function, suggesting a potential, yet underappreciated, involvement of OPCs in the therapeutic mechanisms of these agents. Given the high genetic heritability associated with psychiatric disorders [159], future research should include systematic screening of ion channel gene variants in individuals with a family history of mental illness. This approach may uncover novel mechanistic links between ion channel dysfunction in OPCs and psychiatric disease susceptibility. Integrating genetic, pharmacological, and functional studies of ion channels in OPCs may provide novel insights into the cellular mechanisms underlying psychiatric disorders.

### 4.5. Neuropathic Pain

NP is clinically defined as pain arising as a direct consequence of a lesion or disease affecting the somatosensory system. Chronic neuropathic pain may be triggered by a variety of etiological factors, including chemical insults, physical trauma, infections, or inflammatory dysregulation [160,161]. A double transgenic mouse model (*DTR^fl/fl^*; *Mog-Cre^+ve^*) was generated to selectively ablate oligodendrocytes via diphtheria toxin injection. A marked reduction in MBP immunoreactivity and in the mRNA expression of oligodendrocyte markers such as *Plp* and *Mag* was observed prior to the onset of sensory dysfunction, suggesting that early oligodendrocyte loss and demyelination may contribute to the development of NP [162].

NP is also a frequent and debilitating complication following SCI, which often results in long-term motor and sensory deficits and imposes a substantial socioeconomic burden [163]. SCI involves complex and multifactorial pathophysiological mechanisms, among which white matter damage and oligodendrocyte loss represent key contributors to disease progression [164]. Transplantation of OPCs has demonstrated therapeutic potential in alleviating pain associated with SCI. Studies show that OPC transplantation not only attenuates NP but also promotes remyelination in animal models of SCI [165,166]. However, despite these promising preclinical results, the clinical translation of OPC-based therapies remains challenging due to ethical concerns and the high financial costs associated with human trials. Alternatively, enhancing the recruitment, proliferation, or differentiation of endogenous OPCs may offer a more accessible strategy for promoting remyelination and pain relief [167].

In light of emerging evidence implicating OPC dysfunction in the pathogenesis of chronic pain, modulating OPC ion channel activity to promote endogenous remyelination and glial homeostasis may offer a novel therapeutic avenue for pain-associated neurological disorders. NP is a multifactorial condition involving the complex interplay of various glial cells. Previous studies have demonstrated that ion channels expressed in microglia (e.g., Kv1.3 [168], Cav3.2 [169]) and astrocytes (e.g., Kir4.1 [170]) contribute to both the pathogenesis and treatment of NP. Notably, ion channels have already been recognized as critical pharmacological targets, leading to the development of novel analgesic agents, such as suzetrigine, a selective inhibitor of Nav1.8 [171]. Interestingly, many of these ion channels are also expressed in OPCs, suggesting that OPCs may participate in the initiation and maintenance of pain through mechanisms involving proliferation, differentiation, and myelination.

While ion channels have been extensively studied in neurons and astrocytes in the context of pain, their functional roles in OPCs under pain-related pathological conditions remain largely unexplored. Emerging evidence has implicated OPCs in the pathogenesis of NP, raising the possibility that ion channels expressed by these cells may contribute to nociceptive modulation and represent potential, though yet unvalidated, targets for analgesic intervention.

Notably, structural alterations in CNS myelin have been observed in various pain models [172,173], suggesting a possible link between myelin integrity, OPC function, and pain processing. These findings prompt several important questions: What signaling pathways regulate ion channel activity in OPCs under pathological conditions? Could dysregulation of these pathways underlie the initiation or maintenance of NP? Elucidating how ion channels modulate OPC behavior in the setting of NP may provide novel insights into glial contributions to pain pathophysiology and potentially inform the development of glia-targeted therapeutic strategies.

## 5. Pharmacological Modulation of OPC Ion Channels: Therapeutic Potential

Ion channels expressed in OPCs not only regulate critical physiological processes but also represent promising therapeutic targets for various neurological disorders. Given their pivotal roles in both physiological and pathological contexts, ion channels are particularly amenable to pharmacological intervention. Although some ion channel modulators, such as nimodipine, have demonstrated beneficial effects in animal models, and others like donepezil are already approved for clinical use, further evidence is needed to validate their therapeutic effects specifically via targeting OPCs.

Several ion channel-targeting drugs have been investigated for their potential to modulate OPC function. Nimodipine, a voltage-gated calcium channel blocker, has been shown to promote OPC maturation and enhance remyelination in EAE models [104,105]. Donepezil, an acetylcholinesterase inhibitor widely used in AD, has also been reported to significantly enhance OPC differentiation through mechanisms independent of its cholinesterase inhibition [143]. Another FDA-approved drug, memantine, a non-competitive NMDAR antagonist, protects against glutamate excitotoxicity and has been shown to modulate oligodendrocyte glutamate signaling [133,147]. By partially blocking NMDARs, memantine may mitigate OPC-related glutamatergic dysregulation, which has been implicated in the pathophysiology of AD. These findings suggest that NMDA receptor antagonists may have therapeutic potential in modulating oligodendrocyte function in neurodegenerative conditions.

Despite these promising results, the translational application of OPC-targeting ion channel modulators remains limited. Most existing data are derived from in vitro or preclinical models, and the cell-type specificity of these compounds, especially their selective action on OPCs versus other glial or neuronal subtypes, remains poorly characterized. Additionally, while several ion channels such as TREK-1 and Kir4.1 are known to play non-myelinating roles in OPCs (e.g., regulating BBB integrity and neuron–glia signaling), pharmacological targeting of these channels in disease models is still lacking. For instance, TREK-1 may be involved in OPC-associated vascular interactions and the maintenance of BBB integrity [60,174]. Nevertheless, its specific regulatory mechanisms within OPCs, and the impact of pharmacological modulation—via agonists such as C3001a [175] or inhibitors such as spadin [176]—on OPC behavior and pathological progression, remain poorly understood.

Future studies should aim to validate these targets in disease-relevant animal models and clarify their OPC-specific effects. Developing more selective modulators and delivery systems will be crucial for translating these findings into effective clinical therapies. Moreover, targeting ion channels involved in non-myelinating OPC functions, such as BBB regulation and neuroinflammation, may open new avenues for treating CNS diseases beyond demyelination.

Taken together, pharmacological modulation of ion channels in OPCs represents a novel and underexplored therapeutic strategy to promote remyelination, neurovascular support, and functional recovery in central nervous system disorders. Continued research is essential to bridge the gap between mechanistic insights and clinical translation.

## 6. Conclusions and Outlook

Ion channels expressed in OPCs are increasingly recognized as key regulators of both canonical and non-canonical functions within the CNS. While their roles in OPC proliferation, migration, and differentiation during developmental myelination are well established [11,12,13], growing evidence suggests that these channels also contribute to a range of non-myelinating physiological processes, including extracellular K^+^ buffering, regulation of BBB integrity, and intercellular communication with neurons and glial cells. For instance, Kir4.1 plays a crucial role in maintaining ionic homeostasis and modulating neuronal excitability [33]. In addition, recent studies have revealed that OPCs engage in vascular interactions that contribute to the regulation of BBB integrity, potentially via K2P channels such as TREK-1 [60,174]. Collectively, these findings support a broader conceptualization of OPC ion channels as dynamic modulators of CNS homeostasis, extending well beyond their traditional roles in developmental myelination.

Among various disease contexts, the role of OPC ion channels in MS has garnered the most attention, particularly the dysfunction of Kir4.1. Loss of Kir4.1 in OPCs has been linked to impaired maturation and remyelination failure [62], highlighting its dual importance in both physiological maintenance and pathological progression. Beyond demyelinating conditions, OPCs are increasingly implicated in neurodegenerative diseases such as AD, where they may influence disease dynamics through glial-neuronal interactions and inflammatory signaling. Ion channels are likely key mediators of these interactions, yet their specific roles remain underexplored. Similarly, although ion channels are well-established targets in chronic pain research, the contribution of OPC-specific channels to maladaptive plasticity in pain circuits is only beginning to be understood.

In summary, future studies should aim to systematically elucidate the spatiotemporal expression patterns and functional roles of ion channels in OPCs across developmental stages and disease states. Particular attention should be paid to their involvement in non-myelinating functions, such as BBB modulation, glial communication, and neuron–glia signaling, which may be critical for understanding OPC contributions to CNS pathology beyond demyelination. Technological advances, including single-cell omics, spatial omics [177], and long-time in vivo imaging [178], will be instrumental in driving this field forward. A more nuanced understanding of OPC ion channels may fundamentally reshape our conception of these cells, from passive progenitors to active participants in CNS regulation, with significant implications for therapeutic development across neurological and psychiatric conditions.

## Figures and Tables

**Table 2:** Ion channels of OPCs involved in diseases.

Diseases	Associated Ion Channel	Supporting Experimental/ Clinical Evidence	Potentially Associated Ion Channel	Supporting Evidence for Potential Associations
Multiple Sclerosis	Kir4.1	In MS lesions, a selective loss of Kir4.1 immunoreactivity specifically on oligodendrocytes is evident [88]. OL-expressed Kir4.1 plays a crucial role in maintaining white matter integrity during demyelinating injury [62].	Piezo1	The expression of Piezo1 in the white matter is markedly reduced in the brain affected by multiple sclerosis [89].
	Nav1.2, Nav1.6	Upregulation of Nav1.2 and Nav1.6 has been consistently observed in demyelinated axonal segments within the experimental autoimmune encephalomyelitis (EAE) model [90].		
	NCX3	Deletion of NCX3 resulted in impaired oligodendrocyte myelination and exacerbated the clinical symptoms of EAE [91].		
Traumatic Injury of Central Nervous System	None	None	Cav	Traumatic white matter injury is associated with the influx of calcium ions through Cav expressed on astrocytes and oligodendrocytes surrounding the axons [92].
Amyotrophic lateral sclerosis	Kir4.1	The SOD1G93A rat model has revealed a downregulation and impaired functionality of Kir4.1 in oligodendrocytes [93].	None	None
Alzheimer’s disease	None	None	Piezo1	The specific inhibition of Piezo1 in aging animals significantly promotes the proliferation and differentiation of OPCs [50].
Psychiatric disorders	TMEM63A	TMEM63A deficiency disrupts OPC differentiation and is associated with ASD and ADHD [94].		
	AMPARs	Ketamine attenuates CSDS-induced myelin loss and depression-like behavior by acting on AMPARs expressed in OPCs [95].		
Neuropathic pain	None			

This table summarizes ion channels in OPCs that are either directly implicated or potentially associated with various neurological diseases. The left columns list ion channels with experimental or clinical evidence supporting their involvement in disease-related OPC dysfunction, while the right columns highlight ion channels with emerging or indirect associations.

## Data Availability

Data sharing is not applicable.

## Abbreviations

OPCs	Oligodendrocyte precursor cells	Cav	Voltage-gated Ca^2+^ channel
OLs	Oligodendrocytes	CPZ	Cuprizone
CNS	Central nervous system	NMDARs	*N*-methyl-_D_-aspartate receptors
MS	Multiple sclerosis	LGICs	Ligand-gated ion channels
ALS	Amyotrophic lateral sclerosis	AMPARs	α-Amino-3-hydroxy-5-methyl-4-isoxazolepropionic acid receptors
AD	Alzheimer’s disease	GABA	γ-aminobutyric acid
NP	Neuropathic pain	GABA_A_Rs	GABA_A_ receptors
BBB	Blood–brain barrier	P2XRs	P2X receptors
PDGFRα	Platelet-derived growth factor receptor alpha	KARs	Kainate receptors
NG2	Neuron–glial antigen 2	CLC-2	Voltage-gated chloride channel-2
Olig2	Oligodendrocyte transcription factor 2	NKCC1	Na^+^-K^+^-Cl^−^-co-transporter 1
PDGF	Platelet-derived growth factor	NCX	Sodium calcium exchanger
NSCs	Neural stem cells	LPS	Lipopolysaccharide
A2B5	Cell surface ganglioside epitope	OGD	Oxygen-glucose deprivation
SOX9	SRY-Box Transcription Factor 9	ROS	Reactive oxygen species
O4	Cell surface markers	FDA	Food and Drug Administration
MYRF	Myelin Regulatory Factor	ATP	Adenosine triphosphate
CNP	2′, 3′-cyclic-nucleotide-phosphodiesterase	HCs	Healthy controls
MBP	Myelin basic protein	ADD	Acquired demyelinating disease
MAG	Myelin-associated glycoprotein	EAE	Experimental autoimmune encephalomyelitis
TTX	Tetrodotoxin	TCNSIs	Traumatic central nervous system injuries
Nav	Voltage-gated Na^+^ channels	SCI	Spinal cord injury
RMP	Resting membrane potential	TBI	Traumatic brain injury
K^+^	Potassium	cFPI	Central fluid percussion injury
Kir	Inward rectifying K^+^ channels	rhEPO	Recombinant human erythropoietin
Kv	Voltage-gated K^+^ channels	CCI	Controlled cortical impact
K2P	Two-pore domain K^+^ channels	AChEI	Acetylcholinesterase inhibitor
tMCAO	Transient middle cerebral artery occlusion	nAChR	Nicotinic acetylcholine receptor
GFP	Green fluorescent protein	MDD	Major depressive disorder
PLP	Proteolipid protein	CSDS	Chronic social defeat stress
Pre-OLs	Pre-myelinating oligodendrocytes	PTSD	Posttraumatic stress disorder
Ca^2+^	Calcium	Na^+^	Sodium
